# Effects of Artificial Extraoral Markers on Accuracy of Three-Dimensional Dentofacial Image Integration: Smartphone Face Scan versus Stereophotogrammetry

**DOI:** 10.3390/jpm12030490

**Published:** 2022-03-18

**Authors:** Hang-Nga Mai, Du-Hyeong Lee

**Affiliations:** 1 Institute for Translational Research in Dentistry, Kyungpook National University, Daegu 41940, Korea; mai.hang.nga1403@gmail.com; 2 Department of Prosthodontics, School of Dentistry, Kyungpook National University, Daegu 41940, Korea

**Keywords:** face scan, dental scan, image integration, extraoral marker, accuracy, stereophotogrammetry, smartphone

## Abstract

Recently, three-dimensional (3D) facial scanning has been gaining popularity in personalized dentistry. Integration of the digital dental model into the 3D facial image allows for a treatment plan to be made in accordance with the patients’ individual needs. The aim of this study was to evaluate the effects of extraoral markers on the accuracy of digital dentofacial integrations. Facial models were generated using smartphone and stereophotogrammetry. Dental models were generated with and without extraoral markers and were registered to the facial models by matching the teeth or markers (*n* = 10 in each condition; total = 40). Accuracy of the image integration was measured in terms of general 3D position, occlusal plane, and dental midline deviations. The Mann–Whitney U test and two-way analysis of variance were used to compare results among face-scanning systems and matching methods (α = 0.05). As result, the accuracy of dentofacial registration was significantly affected by the use of artificial markers and different face-scanning systems (*p* < 0.001). The deviations were smallest in stereophotogrammetry with the marker-based matching and highest in smartphone face scans with the tooth-based matching. In comparison between the two face-scanning systems, the stereophotogrammetry generally produced smaller discrepancies than smartphones.

## 1. Introduction

A two-dimensional (2D) photograph of a smiling face and the anterior teeth is a basic way of applying facial infographics to digital smile design [1]. On the basis of facial and dental information in the digital photographs, facial reference lines and preferred tooth shapes can be made through the use of special image processing software [2]. However, since the software enables only 2D image creation, it is difficult to provide three-dimensional (3D) dental information and details of the corresponding facial structure [3,4]. With the rapid development of digital optical scanning technology, it has been possible for noncontact face-scanning devices to produce a 3D replica of human facial soft tissue [5,6]. The 3D approaches have revolutionized face assessment by enabling the generation of a facial digital model that can be coupled with digitized dentition and a 3D radiographic image of underlying bones to build up a complete virtual patient, which in turn enables a comprehensive diagnosis and virtual treatment planning [7,8].

**Figure 1:**
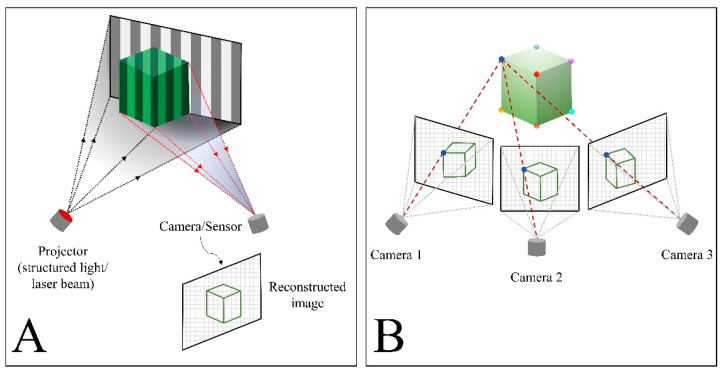
Schematic illustrations of three-dimensional face-scanning technologies. (**A**) Structured light and laser scanner. (**B**) Stereophotogrammetry.

The 3D face-scanning systems can be classified into three major categories: stereophotogrammetry, structured light scanning, and laser scanning [9] (Figure 1). In stereophotogrammetry, multiple single-lens reflex cameras are positioned at a fixed distance from the patient and at a fixed angle on fixation frames to ensure overlapping fields of view [10]. Multiple photos of the patients are taken within milliseconds and imported into dedicated software to reconstruct a 3D image of the face through the use of specific algorithms [11]. Structured light and laser technologies, on the other hand, work by projecting a pattern of light or a laser beam onto the subject and capturing the light reflections with sensors to record the geometry of the surfaces [12,13]. The use of light projection technology enhances the accuracy of facial surface mapping, which results in a higher resolution of the reconstructed models [14]. Infrared structured light depth-sensing cameras have been developed for smartphones [15,16]. This technology enables 3D facial digitization by a smartphone with a dedicated face scan application [17,18,19].

Integration of the digital dental model into the 3D facial image allows the restoration to be modified directly in accordance with the patient’s facial appearance in the computer-aided design stage [20,21,22]. For matching a digital dental model to a 3D facial image, the anterior teeth exposed in a smile on the scanned face are used as reference [23]. To obtain an image of a full set of anterior teeth, the smile or lip position of the patient must be as wide as possible [20]. The accuracy of the dentofacial matching is affected by the capability of the 3D face scans to provide a clear appearance of the anterior teeth [24]. Tooth structure images digitized by 3D face scanners are often deformed due to limitations in scanning morphological characteristics in narrow areas and subsequent faulty image reconstruction. The shininess of the teeth and gingiva also hampers accurate capturing of the intraoral structures [24]. The unclear appearance of the dental structure on the 3D facial models could be problematic for the tooth-based matching method to correctly perform the dentofacial image integrations due to the lack of clear reference landmarks for image matching. To enhance the accuracy of image matching between the 3D face scans and digitized dentition, researchers have used extraoral markers or a transfer jig [20,25,26]. The artificial markers supply distinct reference landmarks that appeared in both the dental and facial images, thereby facilitating the image matching process and enhancing the accuracy of the matching results.

The smartphone 3D face scan is becoming popular in the dental field [27]. Clinical trials for integrating 3D face scans and digital dental models obtained by smartphone are increasing. However, the accuracy of image integration has not been elucidated. The aim of this study was to evaluate how the use of extraoral markers affected the accuracy of integration between 3D face scans obtained by smartphone, in comparison with the results in face scanning by stereophotogrammetry and digital dental models. The first null hypothesis was that the use of extraoral markers would not affect the accuracy of 3D dentofacial integration. The second null hypothesis was that the smartphone and stereophotogrammetry face scanners would not differ with regard to the accuracy of dentofacial image integration.

## 2. Materials and Methods

**Figure 2:**
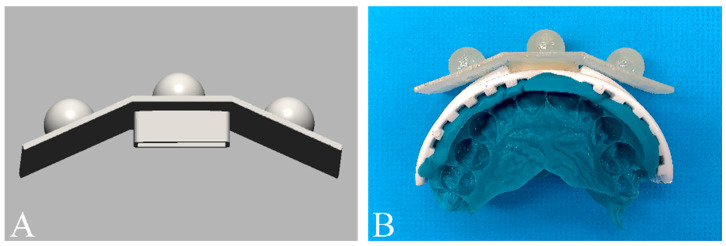
Extraoral marker base. (**A**) Computer-aid-design of the extraoral marker base. (**B**) Attachment of marker base to a disposal bite tray to fabricate a customized transfer jig.

An extraoral marker base was designed using a 3D image computer software (Geomagic DesignX; 3D Systems, Rock Hill, SC, USA) (Figure 2A), and was printed using a digital light processing 3D printer (Meg-Printer II; MegaGen, Daegu, Korea) with a resin material (Raydent C&B; Ray Co., Hwaseong, Gyeonggi-do, Korea). The marker base was attached to a disposable bite tray (Solo Tray P5; Jini Dental, Goyang, Gyeonggi-do, Korea), and an intraoral silicone impression was taken in a volunteer with polyvinyl siloxane (Aquasil Ultra Rigid Regular Set; Dentsply Sirona, Philadelphia, PA, USA) in the maximal intercuspal position (Figure 2B). The volunteer had complete anterior dentition, no facial deformities, and no history of maxillary surgery.

**Figure 3:**
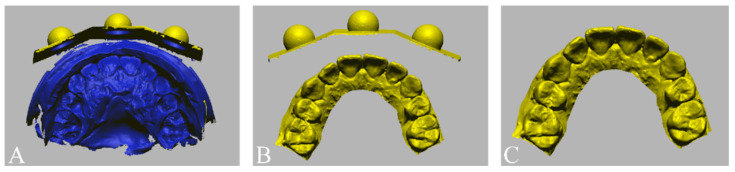
Formation of the digital dental model. (**A**) Scan of the transfer jig with a negative imprint (impression) of the dental arch. (**B**) Conversion of the negative form of the digitized impression image into the positive form using image reversal technique (flip normal) to generate the digital models with markers. (**C**) Deletion of the marker base to make the dental model without the marker.

The bite tray with the extraoral marker base was digitized with the use of a laboratory scanner (IDC S1; Amann Girrbach, Kobach, Austria). To generate digital dental models, the impression of the dental arch was firstly scanned with an intraoral scanner (MEDIT i700; Medit, Seoul, Korea). The digitized impression, which is the negative imprint of the dentate arch, was then reverted to generate the positive form using the “reverse normal” function of the computer software (Geomagic Design X) (Figure 3) [28].

**Figure 4:**
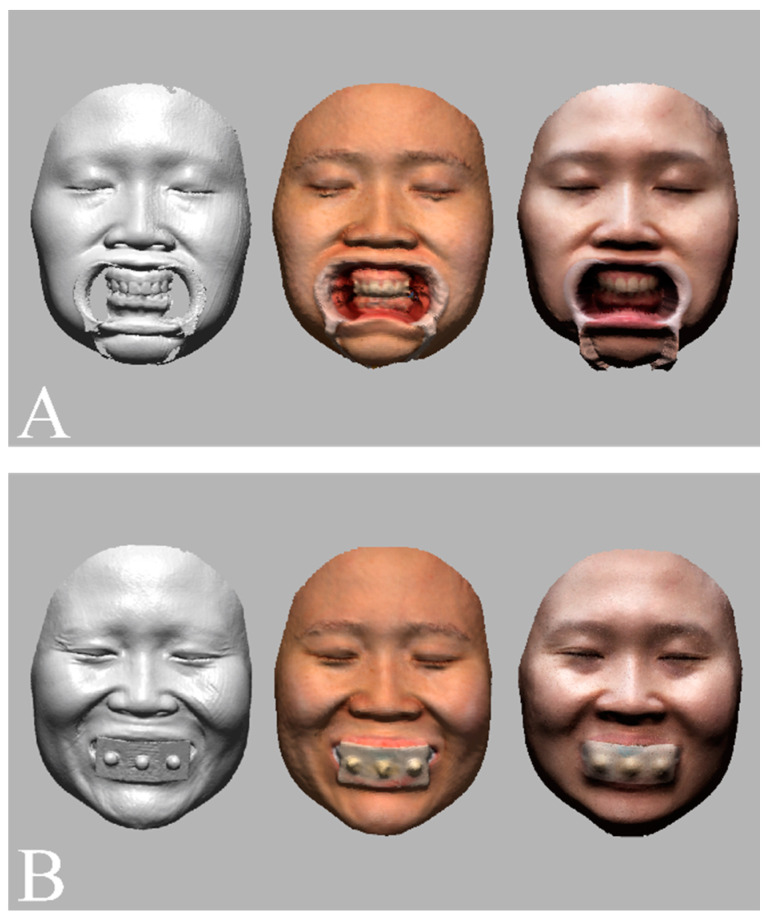
Face scan images obtained by an industrial laser optical scan (left), a stereophotogrammetry (middle), and a smartphone face scan (right). (**A**) Facial models in which cheek retractors were used to fully expose the anterior dentition in occlusion. (**B**) Facial models obtained with the artificial extraoral markers.

Facial images of the volunteer were acquired with three different 3D face-scanning systems: stereophotogrammetry ((Canon EOS 100D with Canon EF LENS 50 mm f1.8 STM; Canon, Tokyo, Japan) and (Di3D capture v.6,8.17.4490, Dimensional Imaging, Glasgow, Scotland, UK)), a smartphone-based structured light scan ((iPhone X; Apple, Cupertino, CA, USA) and (Bellus3D v1.8.6; Bellus3D, Campbell, CA, USA)), and laser scan ((MetraSCAN-R™; Creaform, Levis, QC, Canada) and (VXelements v.7.0.3; Creaform, Levis, QC, Canada)). Before the face was scanned, all facial accessories were removed, and hair that obscured the forehead and ears was pulled up to expose the volunteer’s full face. The face was scanned with the head upright and in the Frankfort horizontal plane parallel to the floor, in accordance with the manufacturer’s instructions. For each face-scanning method, two facial images were obtained, one in which a cheek retractor was used to fully expose the anterior dentition in occlusion (Figure 4A) and one with the bite tray and the marker base attachment (Figure 4B). The scanning protocol was fully explained and written informed consent was obtained before the face was scanned. The 3D facial images were saved in the wavefront object (OBJ) file format. The 3D facial image obtained from the high-end industrial laser scanning system was used as a reference image. Unnecessary areas for image matching, such as head hair, the ears, and the neck were trimmed from the images to enhance the accuracy of image matching [29].

**Figure 5:**
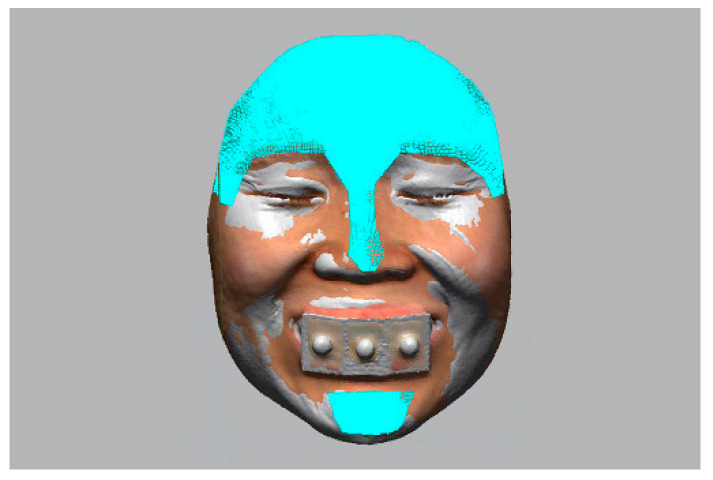
Orientation of facial models into the same coordinate system with the use of immobile face structures as references.

The facial models obtained from stereophotogrammetry and smartphone-based structured light scanning systems were registered to the reference image with the use of the immobile face structures to orient images in the same coordinate system (Figure 5) [30].

**Figure 6:**
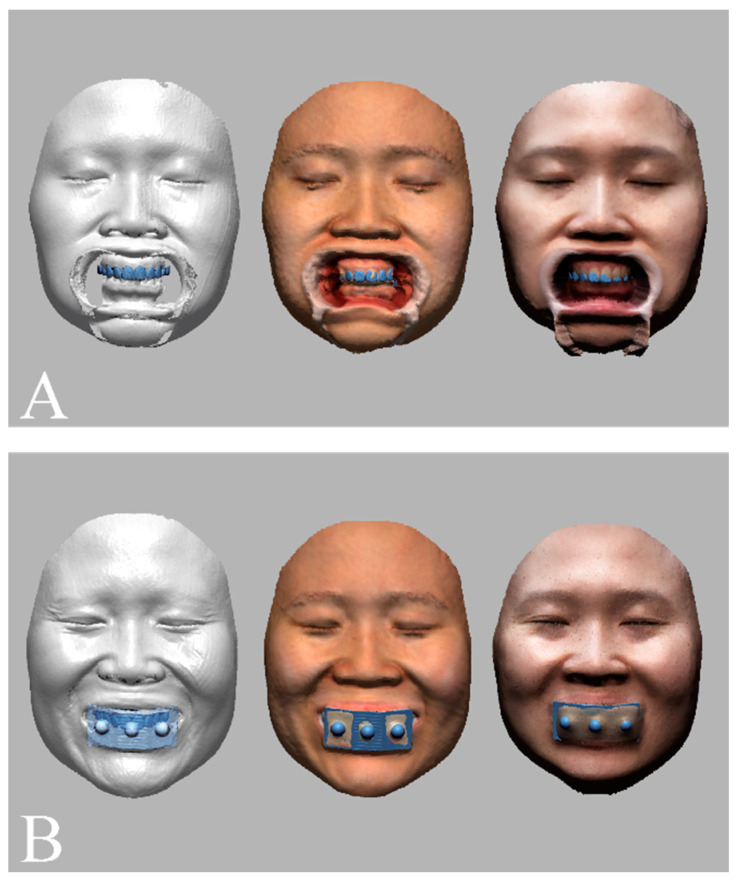
Dentofacial image integration between facial models obtained by an industrial laser optical scan (left), a stereophotogrammetry (middle), a smartphone face scan (right) and dental models and with the use of (**A**) a tooth-based matching method and (**B**) an extraoral marker-based matching method.

The digital dental models were then superimposed on the facial models in four different matching conditions (*n* = 10 per group; total = 40; Figure 6). For the tooth-based matching group, the image was superimposed on the basis of three anatomic landmarks: the interdental papilla of teeth 11 and 21 and the incisal edges of teeth 13 and 23 [24]. For the marker-based matching group, the artificial extraoral markers were used for matching [25]. All image-merging processes were conducted by an examiner who was proficient in 3D image superimposition and familiar with the software (Geomagic DesignX). To avoid the risk of methodological bias, the examiner was kept unaware of the study purpose, each matching process was performed in an individual session, and a two-week interval separated each matching session.

**Figure 7:**
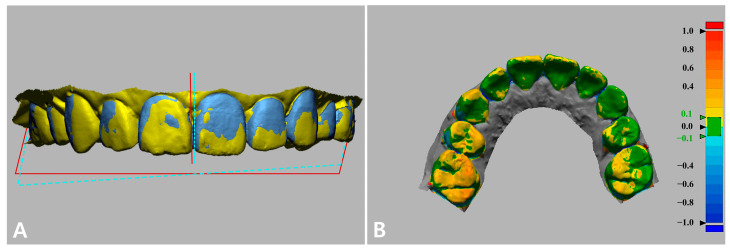
Evaluation of the accuracy of the image integration by measuring (**A**) interincisal midline linear deviation, occlusal plane deviation, and (**B**) 3D surface deviation. Green indicates perfectly matched surfaces (error: ±0.1 mm). Yellow to red shades: the test model was larger than the reference (error: 0.1 mm to 1.0 mm). Light blue to dark blue shades: the test model surface was smaller than the reference (error: −0.1 mm to −1.0 mm).

To evaluate the accuracy of the dentofacial integration, the 3D deviation of matched maxilla models was measured with regard to general 3D positional discrepancy, occlusal plane, and dental midline (Figure 7). The general 3D positional discrepancy of image matching was calculated as root-mean-square errors (**RMSE**) between 3D surfaces with the following equation [31]:

(1) $$RMSE=\sqrt{\frac{\sum_{i=1}^{n}{\left(x_{1,i}-x_{2,i}\right)}^{2}}{n}}$$

where $x_{1,i}$ is the measuring point i on the reference image, $x_{2,i}$ is the measuring point i on the scanned image, and n is the total number of measuring points.

The occlusal plane on the dental model was constructed virtually on the basis of selected points: the incisal edge of the upper central incisors and the mesiobuccal cusps of the first upper right and left molars. The dental midline was identified as the vertical line between the two central incisors.

### Statistical Analysis

All statistical analyses were performed with statistical software (IBM^®^ SPSS^®^ version 25.0; IBM Inc., Armonk, NY, USA). Image discrepancy for each outcome variable was calculated as mean ± standard deviation, and the Mann–Whitney U test was used to compare the results in different face-scanning systems and image-matching methods. The effect of interactions between scanning systems and image-matching methods on the accuracy of image integration was statistically assessed with a two-way analysis of variance. The statistical significance level was set at α = 0.05.

## 3. Results

**Figure 8:**
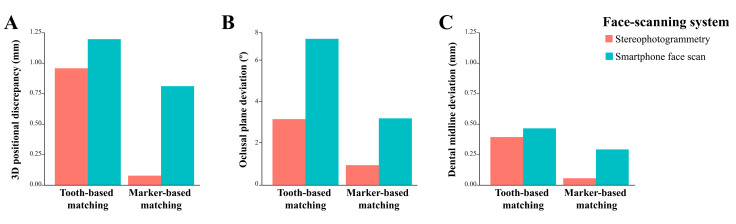
Discrepancy of the image integration. (**A**) Three-dimensional positional discrepancy. (**B**) Occlusal plane deviation. (**C**) Dental midline deviation.

Table 1 and Figure 8 show positional deviations of the integrated dentofacial images. The marker-based matching method resulted in significantly smaller 3D positional discrepancy and occlusal plane deviation than did the tooth-based matching method with both stereophotogrammetry and smartphone face-scanning systems (*p* = 0.008). The marker-based matching method resulted in significantly smaller deviation in midline deviation than did the tooth-base matching method in face scanning by stereophotogrammetry (*p =* 0.032). However, no statistically significant difference was found between the two matching methods in face scanning by the smartphone (*p* = 0.151).

A comparison of the face-scanning systems revealed that the stereophotogrammetry system resulted in smaller deviations than did the smartphone system in all measurements of outcome variables, regardless of the methods used for image matching. The differences in the matching deviation resulting from the two scanning systems were statistically significant except for the outcome of dental midline deviation when the tooth-based matching was used (*p* = 0.690).

The results of the two-way analysis of variance indicated that the matching methods influenced the accuracy of image integration differently according to the different face-scan systems with regard to 3D positional discrepancy (adjusted R^2^ = 0.971), occlusal plane deviation (adjusted R^2^ = 0.969), and dental midline deviation (adjusted R^2^ = 0.437; *p* < 0.001) (Table 2).

## 4. Discussion

According to the results of this study, the use of extraoral markers could improve the accuracy of image matching between 3D facial scans and intraoral scans. Therefore, the first null hypothesis, namely that the use of extraoral markers would not affect the accuracy of 3D dentofacial integration, was rejected. The results corresponded well with those of a previous study [32], in which the use of extraoral markers was an effective and accurate method for dentofacial model registration. Due to the limitations in obtaining a 3D facial model with the clear appearance of dentition, the use of extraoral markers has been suggested to enhance the accuracy of integration between facial models and digital dental models [20,25,26]. The matching between facial scans and intraoral scans is based mostly on the iterative closest point (ICP) algorithm, which requires a clear appearance of objects with sufficient differences in shape features, such as curvature changes, to improve its accuracy significantly [24,30]. Hence, the appearance of teeth that are partially covered by the lips, such as canines and premolars, may be inadequate for image matching; also, accurate matching of teeth with less geometric variation in surfaces, such as incisors, may be difficult [24]. The use of a perioral scan together with an intraoral scan of the dentition to improve the matching accuracy has been reported in a previous study [33]. Accordingly, the inclusion of the perioral structure could provide a larger reference area for image matching. However, the absence of clear landmarks on soft tissues could be problematic for the image stitching process and lead to inaccuracy of the reconstructed scan images. The extraoral markers, in contrast, provide larger protruding landmarks with specific shapes that have clearer appearances on the scanned images, which could improve the accuracy of matching.

In comparison between face-scanning systems, stereophotogrammetry generally exhibited higher accuracy in dentofacial image matching than did the smartphone system. Also, the influence of matching methods on the accuracy of image integration is more significant in stereophotogrammetry than in the smartphone system. Thus, the second null hypothesis, namely that the accuracy of 3D dentofacial integration would not differ between stereophotogrammetry and the smartphone system, was rejected. The reason may be that the errors in the dental and marker areas of the face models differ according to different scanning technologies [34]. In the stereophotogrammetry face scan, a multiphotogrammetry approach bases the 3D facial models on several captured photographs of the face. Thus, the quality of the scans depends largely on the camera setting, and the light reflection while the photo is taken [11]. During image capture, shiny objects, such as teeth, may appear concave in the stereophotogrammetry models as a result of glare [29]. In contrast, the marker, which was fabricated with material that reflected less light, can be photographed with better quality than the teeth.

The smartphone 3D depth camera works by projecting a dot light pattern onto the subject; the dot light reflection is captured with a proximate sensor to build up a depth map of the face, by means of a dedicated smartphone face scan application [27]. In this manner, regions of low depth contrast, such as central incisors, and regions of high depth contrast, such as canines, are captured with perspective distortion in the smartphone face-scanning models due to the inability of the application to analyze atypical depth information [35]. Moreover, as the appearance of a translucent object is significantly affected by the light refraction and transmission, when a high translucent object was scanned by a depth camera, the resultant depth map may present some errors [36]. Thus, the markers fabricated by high opaque materials are recommended. The use of markers may have less effect on enhancing the accuracy of the dentofacial integration in smartphone face-scanning systems than it did in the stereophotogrammetry since the accuracy of the marker images was also significantly influenced by the depth contrast of the marker. Another source of errors may be the motion artifacts that arise during the scanning process [27]. In stereophotogrammetry system, the cameras used to capture the facial image are fixed on stable tripods or frames, and the images are captured with only a single scan that can be conducted in a millisecond, whereas the mobile smartphone camera required movements of the head or the camera during the scanning. Therefore, in comparison with stereophotogrammetry face scans, the images captured by mobile smartphone are more prone to motion artifacts.

In this study, the dentofacial integration was conducted on facial and 3D dental models. The accuracy of smile evaluation has been reported to depend on the use of 2D photographs or 3D models [37]. Accordingly, the dental disparities on the 3D models were evaluated more accurately by dentists than were the same discrepancies in the 2D photographs. One reason may be the influence of the viewing angles on the esthetic ratings of the smile, in as much as the observers preferred higher and centered views of the smile over lower angle views [38]. In the 3D approach, a wide angle of view can be simulated by rotating the 3D models; thus, the smile may receive higher objective ratings than it did in a still 2D image. Another reason why dentists more accurately evaluated discrepancies in the 3D images may be that 2D photographs are more limited in representing the entire occlusal components. Previous studies on smile perception also demonstrated an intimate relationship between the occlusal plane and midline deviation [21,39,40]. Observers probably gave lower esthetic ratings to smiles in which the maxillary midline was altered by occlusal tilting than to smiles in which midline deviations existed without occlusal tilting [39,40]. In comparison with the 3D simulations, in which the spatial relations between the occlusal plane and maxillary midline were represented in three dimensions, the smile evaluations with 2D photographs may be less accurate since only the frontal view of the anterior teeth was shown.

Digital dentofacial integration creates a 3D virtual patient that is useful for simulating the treatment plan and providing effective communications among patients, clinicians, and dental technicians. However, positional deviations of the integrated dentofacial model may lead to an inaccurate esthetic prognosis [41]. Previous studies have reported that the deviations of 2 mm or more of the dentofacial midline, and 4° or more of the occlusal plan are noticeable, and thus, may greatly affect the diagnosis, clinical decision-making, and treatment planning [42,43,44]. It was demonstrated in the present study that the differences in facial scanning and image-matching methods greatly influenced the accuracy of digital dentofacial integration. The midline deviations in all tested conditions were within clinically acceptable threshold values; whereas, when the tooth-based matching method was used in the smartphone face scanning system, the occlusal plan deviation was increased up to 7°. Results of this study may provide the clinicians with some suggestions and awareness for selecting suitable face-scanning systems and matching methods based on the clinical needs.

The limitations of this study were related to the lack of variety in the face and types of extraoral markers, inasmuch as only one person was photographed. The process of 3D face scanning is influenced by the characteristics of the face, such as shape, size, and positions of facial organs. Human faces are diverse; therefore, a large-scale study with numerous participants is recommended to investigate the effects of diversity of facial characteristics on the accuracy of 3D face scans and dentofacial integration. Also, studies on the relation of various extraoral markers and matching accuracy are required in the future. In addition to the influences of the scan objects, possible errors caused by scanning conditions and operators with different experiences should be assessed in further investigations.

## 5. Conclusions

Within the limitations of this study, it could be concluded that the accuracy of dentofacial image integration was significantly enhanced with the use of artificial extraoral markers and stereophotogrammetry face scanning system. Clinical studies with other 3D facial scan applications and devices should be conducted to further the impact of this study results.

## Figures and Tables

**Table 1:** Discrepancy in the dentofacial image integration.

Parameters	Face-Scanning System	Tooth-Based Matching Mean (SD)	Marker-Based Matching Mean (SD)	*p*
Three-dimensional positional discrepancy (mm)	Stereophotogrammetry	0.959 (0.043) ^a,1^	0.078 (0.041) ^b,1^	0.008
	Smartphone face scan	1.197 (0.106) ^a,2^	0.812 (0.065) ^b,2^	0.008
*p*		0.016	0.008	
Occlusal angle deviation (degrees)	Stereophotogrammetry	3.187 (0.147) ^a,1^	0.955 (0.350) ^b,1^	0.008
	Smartphone face scan	7.087 (0.608) ^a,2^	3.225 (0.242) ^b,2^	0.008
*p*		0.008	0.008	
Dental midline deviation (mm)	Stereophotogrammetry	0.393 (0.257) ^a,1^	0.054 (0.074) ^b,1^	0.032
	Smartphone face scan	0.466 (0.031) ^a,1^	0.292 (0.178) ^a,2^	0.151
*p*		0.690	0.032	

Superscript alphabetical letters in the same row indicate a significant difference between matching methods; superscript numbers in the same column indicate a significant difference between face-scanning systems.

**Table 2:** Analysis of between-factors effects on the positional deviations of the digital dental models integrated into the facial images.

Outcome Variable	Source	Sum of Squares	*df*	Mean Square	*F*	p *
Three-dimensional positional discrepancy	Face-scanning system × image-matching method	6.984	3	2.328	440.965	<0.001
Occlusal plane deviation		184.681	3	64.894	408.234	<0.001
Dental midline deviation		0.965	3	0.322	11.102	<0.001

* Two-way analysis of variance. Abbreviation: df, degrees of freedom.

## Data Availability

Not applicable.
